# Altered Immune Profiles of Natural Killer Cells in Chronic Hepatitis B Patients: A Systematic Review and Meta-Analysis

**DOI:** 10.1371/journal.pone.0160171

**Published:** 2016-08-11

**Authors:** Qiong-Fang Zhang, Jian-Ying Shao, Wen-Wei Yin, Yang Xia, Ling Chen, Xing Wang, Huai-Dong Hu, Peng Hu, Hong Ren, Da-Zhi Zhang

**Affiliations:** 1 Key Laboratory of Molecular Biology for Infectious Diseases (Ministry of Education), Institute for Viral Hepatitis, Department of Infectious Diseases, Second Affiliated Hospital, Chongqing Medical University, Chongqing, PR China; 2 Department of Urinary Surgery, First Affiliated Hospital of Chongqing Medical University, Chongqiong, China; 3 Department of Orthopedic Surgery, Second Affiliated Hospital of Chongqing Medical University, Chongqiong, China; The Chinese University of Hong Kong, HONG KONG

## Abstract

**Background:**

Natural killer (NK) cells are the main effective component of the innate immune system that responds to chronic hepatitis B (CHB) infection. Although numerous studies have reported the immune profiles of NK cells in CHB patients, they are limited by inconsistent results. Thus, we performed a meta-analysis to characterize reliably the immune profiles of NK cells after CHB infection, specifically frequency, phenotype, and function.

**Methods:**

A literature search of the computer databases MEDLINE, PUBMED, EMBASE, and Cochrane Center Register of Controlled Trails was performed and 19 studies were selected. The standard mean difference (SMD) and 95% confidence interval (CI) of each continuous variable was estimated with a fixed effects model when I^2^ < 50% for the test for heterogeneity, or the random effects model otherwise. Publication bias was evaluated using Begg’s and Egger’s tests.

**Results:**

The meta-analysis of publications that reported frequency of peripheral NK cells showed that NK cell levels in CHB patients were significantly lower compared with that of healthy controls. A higher frequency of CD56^bright^ NK subsets was found in CHB patients, but the CD56^dim^ NK subsets of CHB patients and healthy controls were similar. CHB patients before and after antiviral therapy with nucleotide analogues (NUCs) showed no statistical difference in NK frequency. The activating receptors were upregulated, whereas inhibitory receptors were comparable in the peripheral NK cells of CHB individuals and healthy controls. NK cells of CHB patients displayed higher cytotoxic potency as evidenced by CD107a protein levels and conserved potency to produce interferon-gamma (IFNγ), compared with their healthy counterparts.

**Conclusion:**

Our results revealed that CHB patients had a lower frequency of NK cells compared with healthy individuals not treatable with antiviral NUC therapy. With an activating phenotype, NK cells in CHB patients showed better cytotoxic potency and conserved IFNγ production.

## Introduction

Hepatitis B virus (HBV) infection is an important health problem worldwide. About 2 billion people have been infected with this virus as reported by the World Health Organization. Over 400 million patients infected with HBV eventually develop chronic hepatitis [1]. Most CHB patients also suffer severe liver disease such as liver cirrhosis and hepatocellular carcinoma [2, 3]. The mechanism by which some HBV patients progress to chronic hepatitis has not yet been fully elucidated [4–6]. The host immune response is considered an important factor for determining whether HBV infection is cleared or persists [7, 8].

NK cells are the main effective population of the innate immune system that responds to viral infection (e.g., HBV) via cytotoxic effectors and cytokine production [9, 10]. NK cells constitute approximately 40% to 60% of liver lymphocytes and 5–15% of total lymphocytes [11, 12]. Derived from hematopoietic progenitor cells in the bone marrow, these large granular lymphocytes have been identified by flow cytometry from CD56 levels and lack of the T-cell marker CD3 (that is, CD3^−^CD56^+^ NK cell status) [13]. CD3^−^CD56^+^ NK cells can be further subdivided into CD56^dim^ NK cells, which express CD16 (Fcγ-receptor) and KIR (killer-cell immunoglobulin-like receptor), and CD56^bright^ NK cells, which lack expression of the two above markers [10, 13]. Although CD56^dim^ NK cells are the largest population and CD56^bright^ NK cells are in the minority in the blood, this subdivision can be significantly changed by persistent viral infection [14].

NK cells display at least two major effector functions to control viral infection: they can directly attack infected cells through cell-to-cell contact, but they also secret a variety of antiviral cytokines such as interferon-gamma (IFNγ) [10, 13, 15]. An increasing number of studies have shown that during HBV infection, effective immune responses by NK cells may lead to the initial control of the acute infection in the early phase and allow the efficient development of an adaptive immune response [16, 17]. Since NK function is closely regulated by activating receptors (NKP30, NKp44, NKp46, NKG2D, NKG2C) and inhibitory receptors (NKG2A, CD158a, CD158b), interactions between NK cell receptors and their corresponding ligands determine the fate of NK cells [15, 18]. Interestingly, in chronic viral infection such as with HBV, NK cell function is impaired through changes in their receptors [15, 19].

The current therapy for CHB is based on the application of pegylated interferon-alpha (Peg-IFNα) or NUCs [20, 21]. Recent studies have reported the effects of anti-viral therapy on innate effectors such as NK cells [22–26]. It has been shown that inhibition of HBV replication by antiviral therapeutic medicine such as NUCs helped to restore partially the function of NK cells in the peripheral blood [22, 23]. However, little is known about the influence of antiviral therapy on the proportion of NK cells.

A large number of studies have addressed the immune profiles of NK cells in CHB patients. Nonetheless, these documented studies are limited by small sample size, differences in patient ethnicities and geographical locations, and, especially, inconsistent results. Therefore, we performed a systematic review and meta-analysis of the currently relevant literature to investigate the frequency, phenotypes, and functions of NK cells in CHB patients.

## Materials and Methods

### Literature search strategy

The study was performed in accordance with the Preferred Reporting Items for Systematic Reviews and Meta-Analyses criteria (PRISMA) (S1 PRISMA Checklist). A search of the MEDLINE, PUBMED, EMBASE, and Cochrane Center Register of Controlled Trails computer databases (from 1980 to December 2015) was performed of manuscripts. The search strategy involved selecting Medical Subject Headings (MeSH) and text words used in combination or alone: “HBV”, “NK cell”, “hepatitis B virus”, “natural killer cells”. The scope of the search was restricted to “human” and “English”. Emails were sent to corresponding authors of related articles in which enough data was not provided. We excluded the non-informative studies if the authors did not reply. Independent searches were conducted by QFZ and JYS.

### Selection criteria

Published studies in English were included when they met the following criteria: randomized control, case-control, or cohort studies; reports of NK cell frequency, phenotypes, receptors, or functions, in peripheral blood or liver tissue; treatment-naïve patients with chronic mono-HBV infection (i.e., no patients taking antiviral therapy or immunosuppressive drugs within 6 months before the sampling); investigating the effect on NK cells of treatment with NUCs or Peg-IFNα. Papers were excluded if they contained unclear or confusing data; or reports of NK cells immune profiles of patients co-infected with hepatitis C virus, hepatitis D virus, or human immunodeficiency virus. The names of the authors or journals of the articles did not influence our selection decisions.

### Data extraction

Two reviewers (QFZ and JYS) independently applied the inclusion criteria, selected the studies, and extracted the data. The following data were extracted from each paper: number of patients in the study; details of the study design; characteristics of patients; treatment regimen; and results measured by flow cytometry. Studies were selected in a 2-stage process. Firstly, the titles and the abstracts from the electronic searches were scrutinized by two reviewers independently (QFZ and JYS) and the full manuscripts of all citations that were likely to meet the predefined selection criteria were obtained. Secondly, final inclusion or exclusion decisions were made upon examination of the full manuscripts. In case of duplicates, the most recent or the most comprehensive publication with all the results was used.

### Study quality

The methodological quality of the articles was assessed using the Newcastle-Ottawa Scale (Wells et al. 2000) [27]. The quality score was calculated on the basis of 3 major components of case-control studies: selection of study groups (0–4 stars), comparability of study groups (0–2 stars), and ascertainment of the outcome of interest (0–3 stars; S1 Table). The quality assessment tool for the cohort study consisted of three domains, including selection of the exposed and unexposed cohort (maximum: 4 stars), comparability of the two cohorts (maximum: 2 stars), and outcome assessment (maximum: 3 stars; S1 Table). A higher score indicated better methodology. The quality of each study was independently assessed by the same two reviewers (QFZ and JYS). In case of disagreement between the 2 reviewers, a third party (DZZ) was consulted.

### Statistical analysis

Statistical analyses for continuous variables were conducted. Heterogeneity was measured using the I^2^ test [28]. In these tests, I^2^ > 50% indicated significant heterogeneity; *P* < 0.05 was also considered to indicate significant heterogeneity. In cases where significant heterogeneity existed, a random effects model was used to quantify heterogeneity across studies. A fixed effects model was used in the other cases [29]. Outcomes were expressed as SMD, with 95%CI. If the value 0 was not included in the 95% CI, the point estimate of the SMD was considered to have reached statistical significance (*P* < 0.05).

To explain heterogeneity among different studies, stratified analyses were performed of alanine transaminase (ALT) levels, HBV-DNA, study location, patient ages, case sample size, and publication year [30]. The ALT levels, immunity status, and NK subsets were further examined in subgroup analyses. Publication bias was evaluated with Begg’s and Egger’s tests [31]. All analyses were performed with STATA version 12.0 (Stata, College Station, TX) in accordance with the recommendations of the manufacturer. *P* < 0.05 was considered significant.

## Results

### Search results and study characteristics

**Fig 1:**
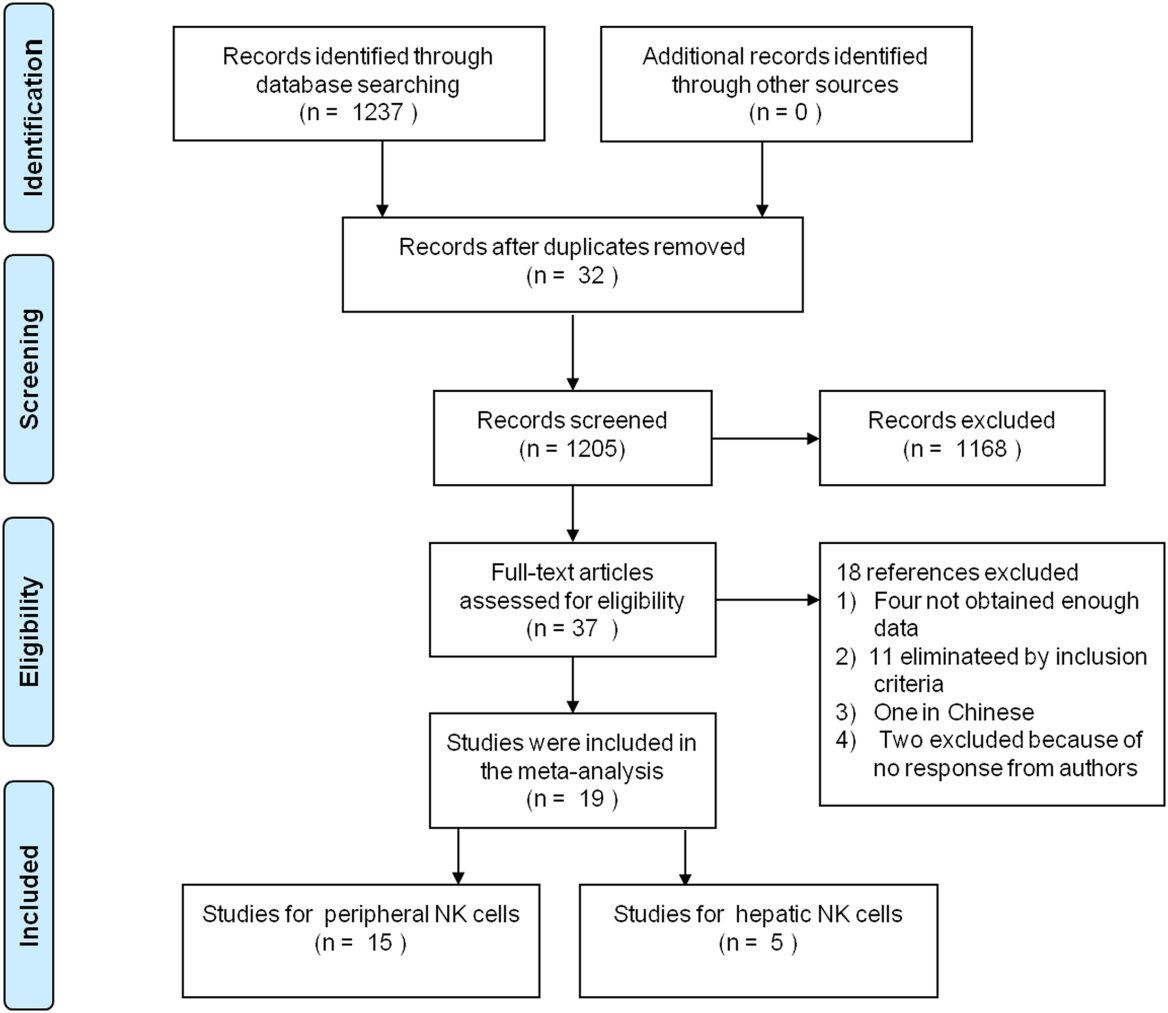
The selection process for eligible studies. Of the 1237 studies initially identified from our electronic search, 19 met the inclusion criteria.

Using the strategy described above, 1237 studies were initially identified and screened for retrieval (Fig 1). After reviewing the title or the abstract, 1205 studies were excluded and 37 were retrieved and subjected to detailed evaluation after removing duplicates and scanning titles and abstracts. Of the 37 studies, one study was excluded because of language. By applying the inclusion and exclusion criteria of the present study, 11 studies were eliminated. Due to lack of data, four studies were excluded, and another two studies were excluded because the author did not respond to requests for information. Finally, 19 studies [14, 22–24, 32–46] (S1 File) comprising 993 patients were included in the meta-analysis.

Of the 19 studies, three were cohort studies and 16 were case control studies. All these studies were published between 2006 and 2015. The basic characteristics of each study are listed in Tables 1 and 2. Eleven of the studies were from China, three from the Netherlands, the one each from Italy, United Kingdom, Ireland, Germany, and France. The sample size of each of the studies ranged from 18 to 154 people. The mean ages ranged from 24.2 to 47.0 years. The male-to-female ratios ranged from 0.7 to 8.0.

**Table 1:** Characteristics of the 18 studies included in the analysis.

First author, Year	Location	Study type	Immune State	Age, y ^a^		Gender, M/F	
				HBV	HC	HBV	HC
Bonorino, 2009	French	Case-control	NA	38 (17–63) ^b^	NA	1.11	NA
Conroy, 2014	Irish	Case-control	NA	34.3 (18–60) ^b^	NA	1	NA
Gu, 2009	Chinese	Case-control	IA	35.50 ± 6.21	35.18 ± 7.01	2.57	2.75
Li, 2012	Chinese	Case-control	NA	34 ^c^	29 ^c^	2.32	1.33
Li, 2014	Chinese	Case-control	IT	28.27 ± 34.59	25.35 ± 40.94	0.88	0.80
	Chinese	Case-control	IA	30.82 ± 68.57	25.35 ± 40.94	4.25	0.80
Li, 2015	Chinese	Case-control	IT	32.8 ± 8.9	42.1 ± 10.9	2.0	1.9
	Chinese	Case-control	IA	38.1± 11.6	42.1 ± 10.9	2.1	1.9
	Chinese	Case-control	IN	32.2 ± 7.4	42.1 ± 10.9	6.3	1.9
Lunemann, 2014	German	Case-control	NA	41 (22–59) ^b^	48 (21–65) ^b^	0.70	1.00
Lv, 2012	Chinese	Cohort	IA	34.4 ± 20.9	35.9 ± 35.0	2.43	5.00
Oliviero, 2009	Italian	Case-control	NA	48 (20–72) ^b^	NA	1.44	NA
Peppa, 2010	English	Case-control	NA	37.2±11.9	30.0 ± 8.5	1.13	1.12
Sprengers, 2006	Dutch	Case-control	NA	36.29±9.84	NA	2.36	NA
Sun, 2012	Chinese	Case-control	NA	33.4± 2.6	34.1 ± 1.8	1.23	1.06
Tjwa, 2011	Dutch	Case-control	NA	38.1 ± 10.1	36.9 ± 8.0	2.08	1.27
	Dutch	Cohort	IA	43.1 ± 12.78	36.9 ± 8.0	2.81	1.27
Tjwa, 2014	Dutch	Case-control	NA	37.15 ± 7.43	0	1.24	NA
Yan, 2006	Chinese	Case control	NA	39.23 ± 18.92	38.17 ± 15.77	1.16	1.80
Zhang, 2011	Chinese	Case-control	IT	24 (16–44) ^b^	27 (20–35) ^b^	1.70	1.89
	Chinese	Case-control	IA	27 (16–46) ^b^	27 (20–35) ^b^	2.79	1.89
Zhao J, 2012	Chinese	Case-control	IA	38 (22–65) ^b^	30 (25–45) ^b^	3.75	3.00
Zhao P, 2012	Chinese	Cohort	NA	27.5 (19–43) ^b^	29 (23–50) ^b^	8.00	3.67
Zheng, 2015	Chinese	Case-control	IT	34 (20–55) ^b^	30 (25–38) ^b^	2.60	1.50
	Chinese	Case-control	IA	31 (17–46) ^b^	30 (25–38) ^b^	1.83	1.50

^a^ Median ± SD, unless noted otherwise;

^b^ median (range);

^c^ median

Abbreviations: ALT, alanine transaminase; IA, immune active; IN, immune inactive; IT, immune tolerant; NA, not available

**Table 2:** Characteristics of the 18 studies included in the analysis.

First author, Year	Immue state	ALT, IU/L ^a^	HBV-DNA ^a^^,^ ^b^	Blood, n		Liver, n	
		HBV	HBV	HBV	HC	HBV	HC
Bonorino, 2009	NA	42.7 ± 27.8	45 ± 1.9 ^c^	19	18	6	0
Conroy, 2014	NA	33.8 (8–143) ^d^	2.92 (0.85–8.65) ^d^	66	62	0	0
Gu, 2009	IA	403.72 ± 258.06	5.85 ± 1.08	100	30	0	0
Li, 2012	NA	81 ^e^	5.27 (3–8.48) ^d^	73	35	0	0
Li, 2014	IT	22.22 ± 27.58	7.58 ± 0.81	15	18	0	0
	IA	199.85 ± 1250	6.62 ± 1.17	42	18	0	0
Li, 2015	IT	<50	7.0 ± 1.8	24	20	0	0
	IA	198.8 ± 113.4	6.2 ± 1.9	40	20	0	0
	IN	<50	<3	22	20	0	0
Lunemann, 2014	NA	30 (14–166) ^d^	3.47 (2.53–7.52) ^c^^,^^d^	17	30	0	0
Lv, 2012	IA	124.5±211.4	7.6 ± 3.2 ^c^	24	12	0	0
Oliviero, 2009	NA	50.5 (13–291) ^d^	5.17 (2.23–8.14) ^c^^,^^d^	22	30	0	0
Peppa, 2010	NA	69.3 ± 99.4	4.4 ± 2.2 ^c^	64	31	8	0
Sprengers, 2006	NA	153.85 ± 189.84	5.67 ± 1.66	47	0	47	0
Sun, 2012	NA	98 ± 117	>3.30 ^c^	154	95	0	0
Tjwa, 2011	NA	60 ± 82.22	6.5 ± 1.9	40	25	0	0
	IA	75 ± 13	7.7 ± 2.3	15	25	0	0
Tjwa, 2014	NA	85.05 ± 64.86	6.0 ± 2.2 ^c^	56	0	56	0
Yan, 2006	NA	369.26 ± 238.87	NA	54	14	0	0
Zhang, 2011	IT	23 (12–26) ^d^	8.43 (7.15–8.81) ^c^^,^^d^	27	26	15	12
	IA	196 (41–1298) ^d^	8.29 (4.40–9.29) ^c^^,^^d^	51	26	29	12
Zhao J, 2012	IA	242 (42–1298) ^d^	7.2 (2.7–8.6) ^c^^,^^d^	19	16	0	0
Zhao P, 2012	NA	165 (12–914) ^d^	4.3 (1.86–9.3) ^d^	18	14	0	0
Zheng, 2015	IT	28 (14–46) ^d^	5.32 (3.00–7.88) ^d^	36	10	0	0
	IA	149 (52–1733) ^d^	5.61 (4.10–8.58) ^c^^,^^d^	34	10	24	0

^a^ Median ± SD, unless noted otherwise;

^b^ log_10_ copies/mL, unless noted otherwise;

^c^ log_10_ IU/mL;

^d^ median (range);

^e^ median

Abbreviations: ALT, alanine transaminase; IA, Immune active; IN, Immune inactive; IT, Immune tolerant; NA, not available

### Peripheral NK cells in CHB patients compared with healthy controls

**Fig 2:**
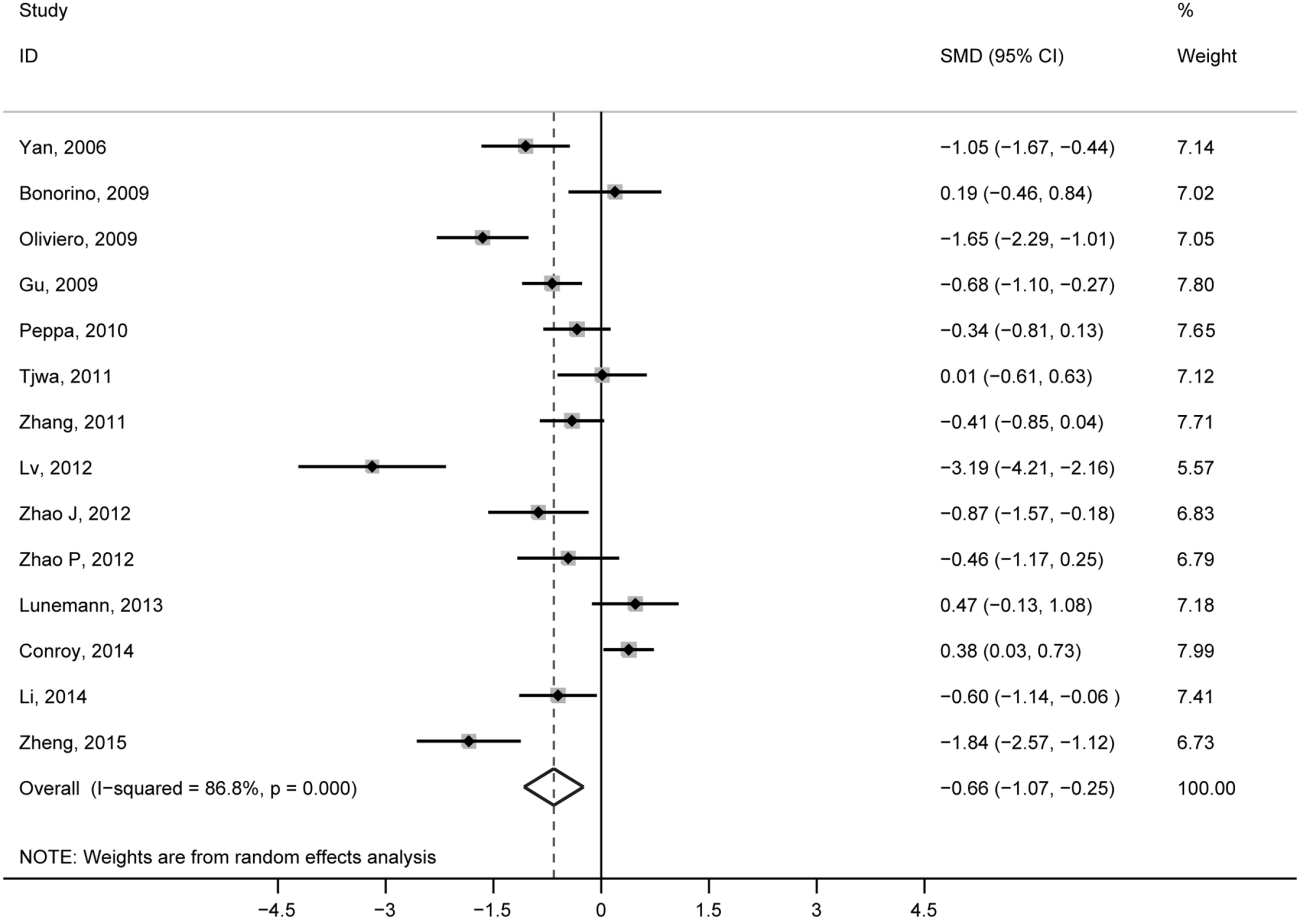
Pooled comparison of peripheral NK cells in CHB patients and healthy controls.

The meta-analysis of the 14 studies showed that peripheral NK cell levels in CHB patients were significantly higher than in the healthy controls (SMD = –0.66, 95% CI: –1.07 to –0.25, *P* = 0.002; Fig 2). There was evidence of high statistical heterogeneity among the studies (I^2^ = 86.8%), and the randomized-effects model was applied. A meta-regression analysis was conducted to examine the source of heterogeneity (S2 Table), and ALT levels had a potency effect on NK percentage (*P* = 0.037).

Based on results from the meta-regression analysis, we subsequently performed a subgroup analysis based on ALT levels (Table 3). Our findings diverged significantly due to the ALT levels. In the subgroup with CHB patients (<80 IU/L), the frequency of NK cells did not change significantly compared to healthy counterparts (SMD = 0.15, 95% CI: –0.16 to 0.46, *P* = 0.355, I^2^ = 44.2%). In the other two subgroups, lower levels of NK cells were found for CHB patients compared with the healthy controls (80–300 IU/L: SMD = –1.46, 95% CI: –2.30 to –0.62, *P* = 0.001, I^2^ = 88.7%) and (>300 IU/L: SMD = –0.76, 95% CI: –1.04 to –0.47, *P* = 0.000, I^2^ = 0.0%). Additionally, the I^2^ statistic (I^2^ = 88.7%) only showed high heterogeneity in the subgroup that consisted of CHB patients with ALT levels 80–300 IU/L.

**Table 3:** Results of subgroup analysis evaluating the difference in NK cells between CHB patients and healthy controls.

NK cells in PBMC, %	Subsgroup	CHB/HC, n	Study no.	SMD	I^2^	95% CI	*P*
Total		616/328	14	–0.66	86.8%	–1.07 to –0.25	0.002
ALT, IU/L	<80	174/158	5	0.15	44.2%	–0.16 to 0.46	0.355
	80–300	298/143	5	–1.46	88.7%	–2.30 to –0.62	0.001
	>300	191/74	4	–0.76	0.0%	–1.04 to –0.47	0.000
Immune state	Immune tolerant	76/54	3	–0.53	72.4%	–1.25 to 0.20	0.153
	Immune active	253/96	5	–0.97	85.5%	–1.23 to –0.72	0.000
NK subsets	CD56 dim	117/130	4	0.08	0.0%	–0.17 to 0.33	0.534
	CD56 bright	119/95	4	0.33	48.4%	0.05 to 0.61	0.021

After further exclusion of studies that contained no relevant information, we performed two subgroup analyses of immunity status (immune tolerant and immune active) and NK subsets (CD56^dim^ NK and CD56^bright^ NK; Table 3). Interestingly, the analysis of studies of immune tolerant patients was non-significant (SMD = –0.53, 95% CI: –1.25 to 0.20, *P* = 0.153) whereas patients in immune active phase showed significantly decreased NK frequency compared to healthy controls (SMD = –0.97, 95% CI: –1.23 to –0.72, *P* = 0.000). Regarding the NK subsets, the frequency of CD56^dim^ NK cells were similar between the CHB patients and healthy controls (SMD = 0.08, 95% CI: –0.17 to 0.33, *P* = 0.534), whereas CD56^bright^ NK subsets displayed a higher frequency in CHB patients compared to healthy controls (SMD = 0.33, 95% CI: 0.05 to 0.61, *P* = 0.021).

A meta-analysis was conducted of four studies that evaluated the NK frequency of CHB patients in liver and peripheral blood (S1A Fig). In the liver, the NK cell populations were larger compared with that in the peripheral blood. When a meta-analysis was performed that excluded these two studies as sources of heterogeneity (S1B Fig) identified by Galbraith’s plots [33, 45], the heterogeneity disappeared (S1C Fig). We also analyzed the role of treatment with NUCs on the frequency of peripheral NK cells (S2 Fig). No significant difference was found in CHB patients before and after treatment.

### NK cell receptors between CHB patients and healthy controls

We also analyzed NK cell receptor expression between CHB patients and healthy controls, including the activating receptors NKp44, NKp46, NKp30, NKG2D and NKG2C, and the inhibitory receptors NKG2A, CD158a, and CD158b (Table 4). Activating receptors NKp46, NKp30, NKG2D and NKG2C were increased, whereas inhibitory receptors NKG2A, CD158a, and CD158b were comparable in the peripheral blood of CHB patients, compared with that of healthy controls. Thus, NK cells displayed an activating phenotype during CHB infection.

**Table 4:** Results of meta-analyses of studies evaluating the difference in NK receptors between CHB patients and healthy controls.

NKR in NK cells, %	CHB/HC, n	Study no.	SMD	I^2^	95% CI	*P*
NKp44	288/139	5	0.137	89.80%	-0.58 to 0.85	0.707
NKp46	288/139	5	0.386	79.90%	0.18 to 0.60	0.000
NKp30	365/169	7	0.585	79.70%	0.13 to 1.04	0.011
NKG2D	172/95	5	0.465	59.70%	0.03 to 0.90	0.035
NKG2C	267/133	6	0.522	94.90%	0.29 to 0.76	0.000
NKG2A	376/174	8	0.002	12.90%	–0.20 to 0.21	0.985
CD158a	208/103	5	-0.072	68.70%	–0.53 to 0.38	0.756
CD158b	235/106	6	-0.079	40.50%	–0.40 to 0.24	0.626

### Production of CD107a and IFNγ in CHB patients compared with healthy controls

**Fig 3:**
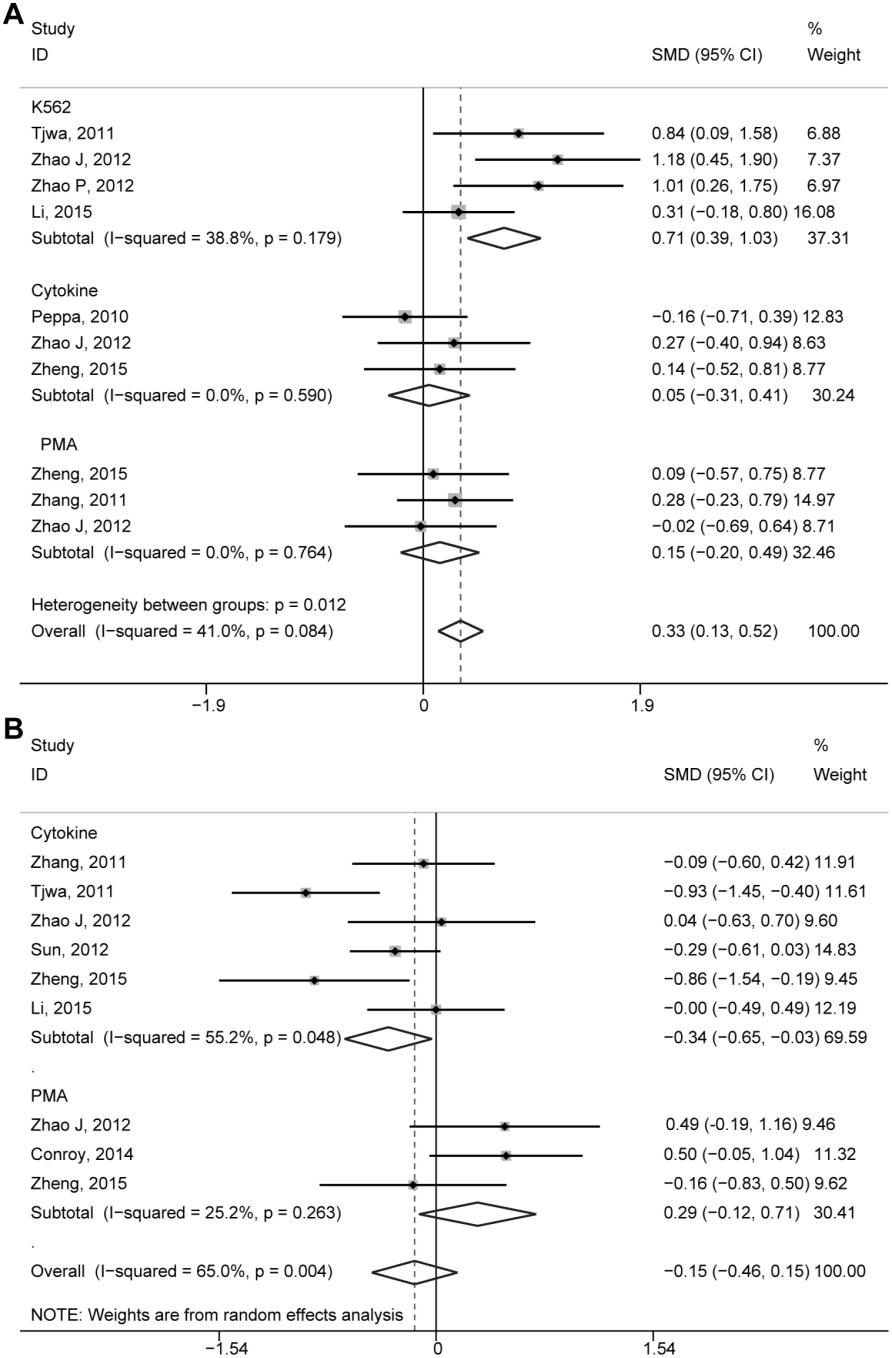
Comparison of CD107a and IFNγ production of peripheral NK cells of CHB patients and healthy controls. (A) Comparison of CD107a production of peripheral NK cells of CHB patients and healthy controls. (B) Comparison of IFNγ production of peripheral NK cells of CHB patients and healthy controls.

We evaluated the cytotoxicity of NK cells and IFNγ production to evaluate the function of NK cells in CHB patients relative to that of the healthy controls. A CD107a degranulation assay was performed as an indirect reflection of cytotoxicity, because it is now widely used to assess the cytotoxic potency of CD8 T-cells and NK cells [47, 48]. Seven studies reported CD107a degranulation of peripheral NK cells stimulated by major histocompatibility complex-devoid K562 target cells, cytokines, or mitogenic phorbol myristate acetate (PMA)/ionomycin. These studies included 400 CHB patients and 159 healthy controls. The total degranulation ability of NK cells was higher in CHB patients compared with the healthy controls (SMD = 0.33, 95% CI: 0.13 to 0.52, *P* = 0.001, I^2^ = 41.0%) and the fixed-effects model was applied (Fig 3A). This difference was more pronounced for peripheral NK cells stimulated by K562 cells (SMD = 0.71, 95% CI: 0.39 to 1.03, *P* = 0.000, I^2^ = 38.8%). However, the differences with healthy controls were not significant when NK cells were stimulated either with cytokines (SMD = 0.05, 95% CI: –0.31 to 0.41, *P* = 0.781, I^2^ = 0.0%) or PMA/ionomycin (SMD = 0.15, 95% CI: –0.20 to 0.49, *P* = 0.406, I^2^ = 0.0%). Seven studies, which included 480 CHB patients and 204 healthy controls, reported IFNγ production of peripheral NK cells stimulated by cytokines or PMA/ionomycin. Our data revealed that IFNγ production of NK cells was comparable in CHB patients compared with that in healthy controls (SMD = –0.15, 95% CI: –0.46 to 0.15, *P* = 0.323, I^2^ = 65.0%; Fig 3B). In the subgroup in which NK cells produced IFNγ in response to cytokines, NK cells in the CHB patients produced less IFNγ compared with the healthy controls (SMD = –0.34, 95% CI: –0.65 to -0.03, *P* = 0.032, I^2^ = 55.2%). Between these groups, IFNγ production was similar after simulation with PMA/ionomycin (SMD = –0.15, 95% CI: –0.12 to 0.71, *P* = 0.166, I^2^ = 25.2%). Taken together, our meta-analysis showed a functional dichotomy in CHB patients as their cytotoxic potency appeared to be elevated, whereas IFNγ secretion, an important non-cytolytic mechanism of virus control, was conserved.

### Publication bias

Begg’s and Egger’s tests were performed to assess the publication bias of the literature, and no evidence of publication bias was found (S3 Table).

## Discussion

Our present work is the first attempt to review the literature and provide a comprehensive and extensive estimate of abnormal immune profiles of NK cells in CHB patients. We demonstrated that NK cells in CHB patients with a lower frequency displayed an active phenotype and exhibited a functional dichotomy featured by an increased cytotoxicity and a conserved cytokine production.

Concomitant with data reported in previous studies [36, 43], our meta-analysis suggests that, among CHB patients, there is a higher proportion of NK cells in the liver than in the peripheral blood. Zhang et al. [36] reported that decreased frequency of hepatic NK cells of CHB patients in immune active phase displayed an activated phenotype, and skewed toward cytolytic activity, but without a concomitant increase in IFNγ production, compared with healthy subjects. Since there is little information in the literature regarding hepatic NK cells, the differences in intrahepatic NK cells between CHB patients and healthy individuals has not been established. Despite the differences in the proportion and functional characteristics of NK cells in the liver and peripheral blood, persistent HBV infection can significantly influence peripheral NK cells, which can mirror alterations in intrahepatic NK cells [49]. Thus, most publications have assessed circulating NK cells, as these are more accessible and also easy to evaluate.

Our meta-analysis revealed that the frequency of circulating NK cells was lower in CHB patients compared with the healthy controls. Noticeably, the NK cell frequency in patients with minimal or no inflammation (ALT < 80 IU/L) or in immune-tolerant phase was comparable to that in healthy controls. This phenomenon might be a reflection of the time required to develop an effective and accurate immune response, from innate to adaptive immunity. In the early stage of HBV infection, the innate immune response is relatively strong. However, HBV-specific immune response is inefficient with anergy, deletion, and altered maturation of HBV-specific effector cells [50]. As a major component of the innate immune system, NK cells dominate the immune effector cell population in this phase and the distribution of NK cells resembles that seen in the normal liver [51]. With the development of adaptive immunity, the innate immune response is reduced [42].

The number of intrahepatic NK cells in immune tolerant phase is putatively higher than that during the immune active phase [33], further supporting the hypothesis above. Our data also confirm a significant reduction in the NK cells of CHB patients in the immune active phase, even though many of the eligible studies lacked the absolute number of NK cells required to perform a meta-analysis. The main reasons for the reduction in NK cells may be because, firstly, in HBV infections under proinflammatory conditions, NK cells are more susceptible to apoptosis [52]. In addition, the clear differences in NK cell frequencies strongly support that HBV itself, like the hepatitis C virus, may be able to significantly suppress the proliferation of NK cells [53–55]. Moreover, the reduced frequency of NK cells may result from the expansion of other cells, such as an increased number of dendritic cells, regulatory T-cells, and T helper 17 (Th17) cells [56–59].

As for NK subsets, increased frequency of CD56^bright^ NK cells in CHB patients but no significant difference was found in CD56^dim^ NK subsets. It is tempting to speculate that persistent HBV infection not only influences the frequency of peripheral NK cells but also modulates these subsets.

The present guidelines support both NUCs and Peg-IFN-α as first-line treatment options [20]. However, a satisfactory antiviral response has been achieved only in a minor population of patients treated with Peg-IFN-α, and the off-treatment durability of response to NUCs is generally low [20, 25, 26]. Since the immune response to CHB infection acts as a determinant of disease prognosis, a better understanding of the immune effect of anti-viral therapy is urgently needed. Despite the influence on the adaptive immune system, the effects of anti-viral therapy on innate effectors such as NK cells remain a strong concern [22–26]. Our results revealed that frequency of NK cells could not be adequately reversed by treatment with nucleotide inhibitors, although some studies have reported functional changes in NK cells [8, 9, 22, 38]. It has been previously shown that Peg-IFN-α therapy could drive the proliferation and expansion, in absolute numbers, of CD56^bright^ NK cell numbers [26]. Tan et al. [25] reported that combined treatment of Peg-IFN-α and oral NUCs has a synergistic effect on innate parameters, such as NK cells, in CHB patients. To improve the therapeutic options for HBV, we need to explore further the immune basis by which HBV impairs anti-viral immune responses.

NK cells are essential effectors of the antiviral response in innate immunity, via the direct killing of infected cells, and produce a variety of antiviral and immunoregulatory cytokines. IFNγ is one of the main cytokines [10, 13, 15]. Furthermore, the functions of NK cells depend on a fine balance between activator and inhibitory receptors [15, 18, 60, 61]. Studies exploring the role of NK cells in persistent HBV infection in recent years have reported inconsistent results. Our results show CHB patients with a predominantly activating phenotype, featuring a higher percentage of NK cells expressing the activating receptors and a similar percentage of NK cells expressing the inhibitory receptors, compared with healthy controls. Because functional changes do not necessarily reflect altered function [62, 63], we subsequently analyzed the cytolytic potency of NK cells and cytokine production in CHB patients. Unexpectedly, in the present study NK activation did not induce all the effector cytotoxic functions of NK cells to an equal degree. NK function was characterized by enhanced cytolytic potency and conserved cytokine production. The results differed according to the stimulation applied to the NK cells. K562 was associated with increased levels, whereas cytokine or PMA/ionomycin resulted in CD107a levels in CHB patients similar to that of the healthy controls. NK cells produced less IFNγ after stimulation with cytokines compared with healthy controls. However, these results did not reach statistical significance when NK cells were stimulated with PMA/ionomycin.

In summary, our results suggest the existence of a selective defect in NK function. It is likely that the elevated NK cytolytic activity could contribute to liver injury, whereas concomitant inefficient IFNγ production may favor viral persistence, further promoting the progression of HBV infection. Such divergence of NK function is in accord with the recent finding that cytokines are trafficked and secreted by completely different pathways to cytotoxic granules in NK cells [64, 65]. And interleukin-10 (IL-10) and transforming growth factor-β (TGF-β) might participate in the pathways [22].

Nonetheless, one should be cautious when explaining these results due to the limitations of the included studies. A limitation in this meta-analysis is the failure to collect enough data regarding hepatic NK cells. We were unable to estimate the alteration of hepatic NK cells in CHB patients relative to healthy individuals. In addition, the optimal way to evaluate the immune profiles of NK cells in CHB patients is to compare CHB patients in each immune phase with healthy controls. Unfortunately, many of the eligible studies in this meta-analysis lacked the necessary information to perform these types of subgroup investigations. Thus, further high-quality studies are still needed to confirm these results.

## Conclusions

The present meta-analysis revealed a lower frequency of NK cells with an activating phenotype in CHB patients. The functional dichotomy of NK cells was characterized by an enhanced cytotoxic potency and a conserved cytokine production, which may be an important mechanism contributing to liver injury and HBV persistence. Our meta-analysis draws a more precise estimation of the altered immune profiles of NK cells during CHB infection. This may further understanding of the mechanism of HBV persistence, and provide an insight into the challenge of our battle against hepatitis B infection for the future.

## Supporting Information

S1 Fig Comparison of hepatic NK cells VS peripheral NK cells in CHB patients. (A) Comparison of hepatic NK cells VS peripheral NK cells in CHB patients with high heterogeneity; (B) Galbraith´s plots for publication heterogeneity for NK cells in liver VS in blood of CHB patients; (C) Comparison of hepatic NK cells VS peripheral NK cells in CHB patients without significant heterogeneity.
(DOC)

S2 Fig Comparison of peripheral NK cells in CHB patients before and after treatment with NUCs. (DOC)

S1 File Included studies in the meta-analysis. (DOCX)

S1 PRISMA Checklist (DOC)

S1 Table Assessment of study quality. (DOC)

S2 Table Meta-regression analysis on 14 selected studies of peripheral NK cells. (DOC)

S3 Table Publication bias were qualitatively assessed by Begg’s and Egger’s tests. (A) Quantitative Data of pooled frequency of peripheral NK cells on Bias. (B) Quantitative Data of pooled frequency of CD107a degranulation of NK cells on Bias. (C) Quantitative Data of pooled frequency of IFNγ production of NK cells on Bias.
(DOC)
