# Supplementary material for: Altered Immune Profiles of Natural Killer Cells in Chronic Hepatitis B Patients: A Systematic Review and Meta-Analysis
Source: PLoS One. 2016 Aug 11;11(8):e0160171. doi: 10.1371/journal.pone.0160171 (PMC4981347; doi:10.1371/journal.pone.0160171)

**S1 Fig.** **Comparison of hepatic NK cells VS peripheral NK cells in CHB patients.** (A) Comparison of hepatic NK cells VS peripheral NK cells in CHB patients with high heterogeneity; (B) Galbraith´s plots for publication heterogeneity for NK cells in liver VS in blood of CHB patients; (C) Comparison of hepatic NK cells VS peripheral NK cells in CHB patients without significant heterogeneity.


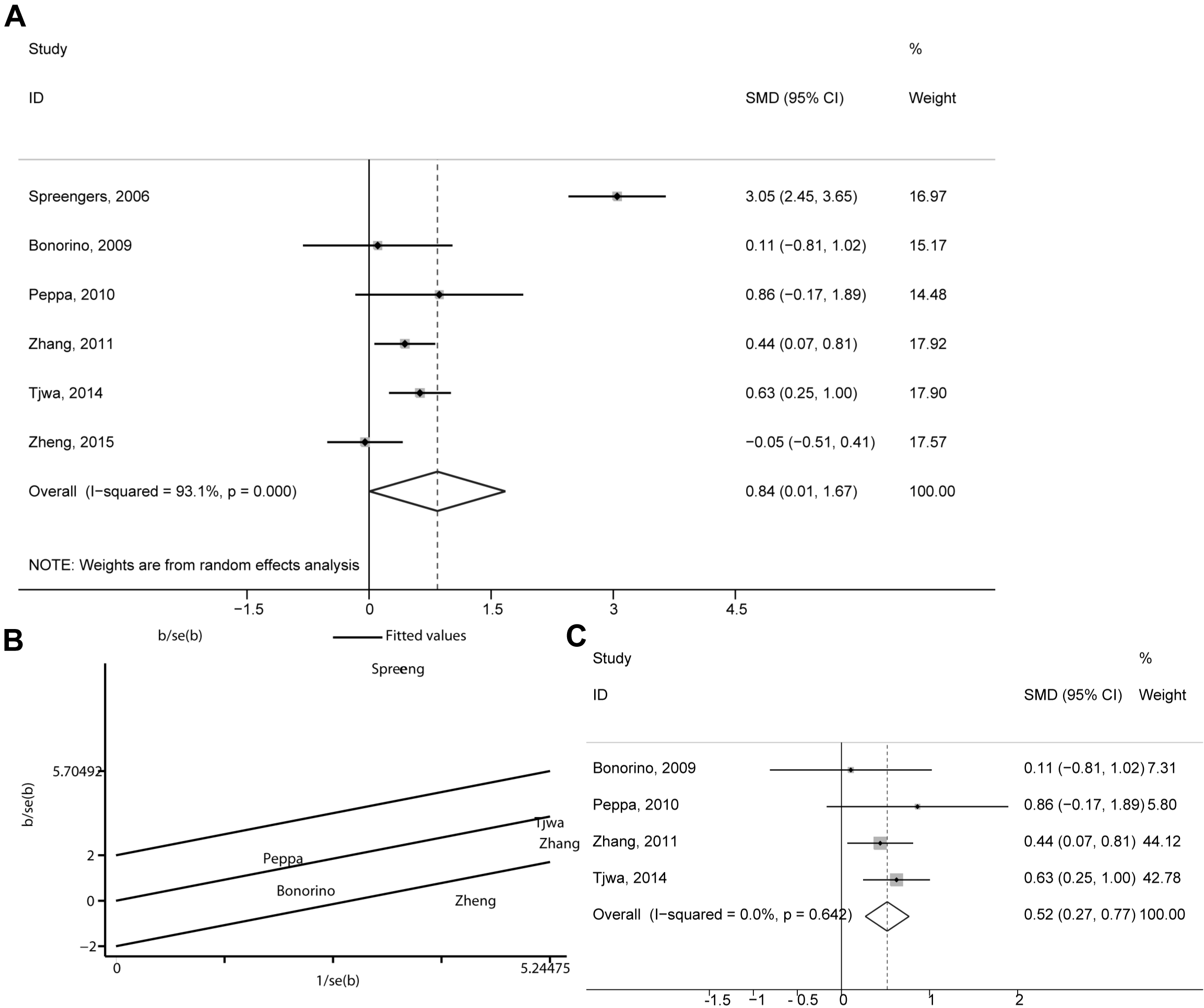

Supplement: S1 Fig — (A) Comparison of hepatic NK cells VS peripheral NK cells in CHB patients with high heterogeneity; (B) Galbraith´s plots for publication heterogeneity for NK cells in liver VS in blood of CHB patients; (C) Comparison of hepatic NK cells VS peripheral NK cells in CHB patients without significant heterogeneity. (DOC) [file pone.0160171.s001.doc]
