# Supplementary figures and images for: Altered Immune Profiles of Natural Killer Cells in Chronic Hepatitis B Patients: A Systematic Review and Meta-Analysis
Source: PLoS One. 2016 Aug 11;11(8):e0160171. doi: 10.1371/journal.pone.0160171 (PMC4981347; doi:10.1371/journal.pone.0160171)

**S2 Fig.** Comparison of peripheral NK cells in CHB patients before and after treatment with NUCs.


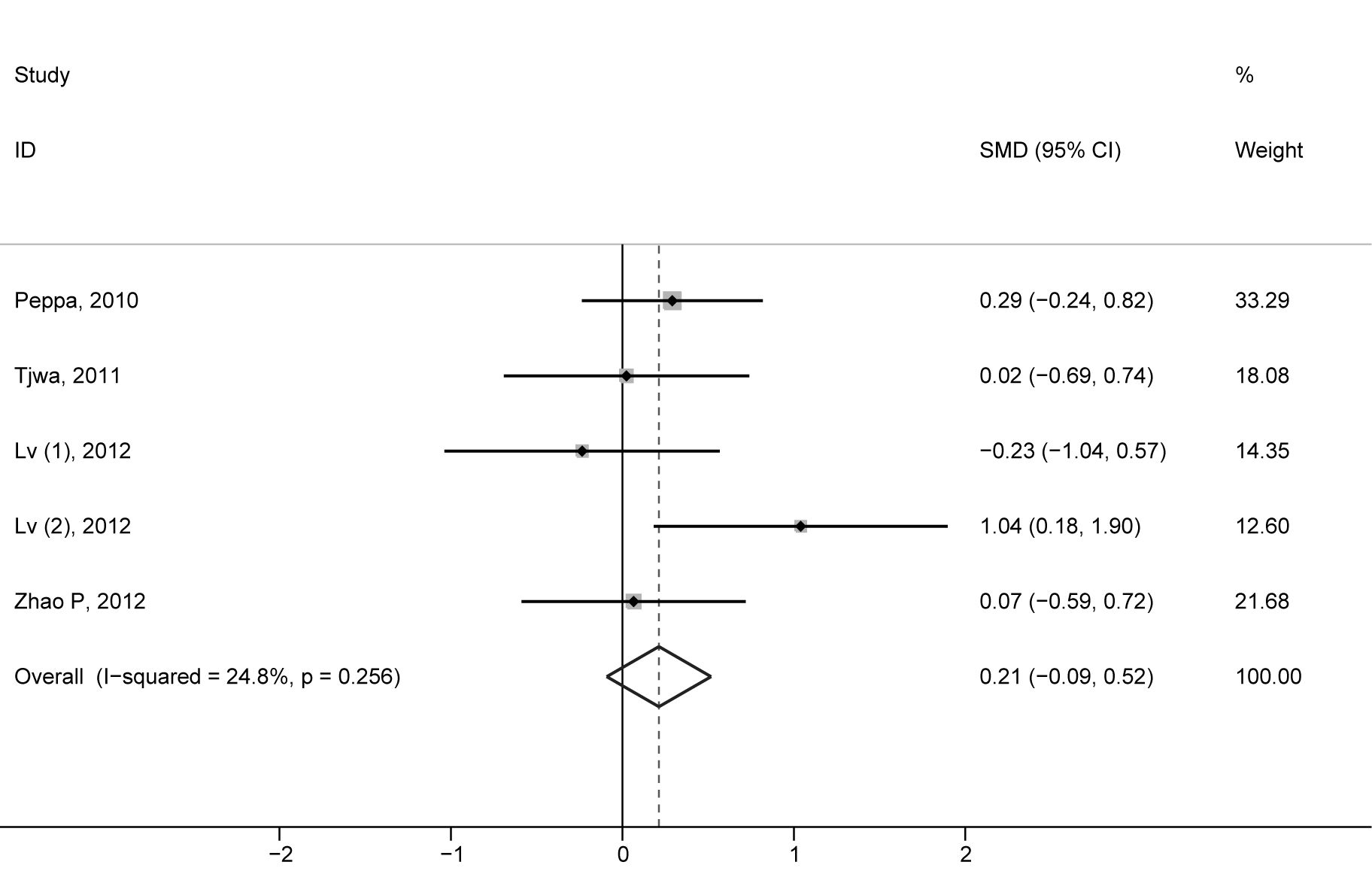

Supplement: S2 Fig — (DOC) [file pone.0160171.s002.doc]
