# Supplementary material for: Altered Immune Profiles of Natural Killer Cells in Chronic Hepatitis B Patients: A Systematic Review and Meta-Analysis
Source: PLoS One. 2016 Aug 11;11(8):e0160171. doi: 10.1371/journal.pone.0160171 (PMC4981347; doi:10.1371/journal.pone.0160171)
Supplement: S1 File — (DOCX) [file pone.0160171.s003.docx]

**Appendix S2. Lists of included studies**

1. Oliviero B, Varchetta S, Paudice E, Michelone G, Zaramella M, Mavilio D, et al. Natural killer cell functional dichotomy in chronic hepatitis B and chronic hepatitis C virus infections. Gastroenterology. 2009;137(3):1151-60, 60 e1-7. Epub 2009/05/28. doi: 10.1053/j.gastro.2009.05.047. PubMed PMID: 19470388.

2. Peppa D, Micco L, Javaid A, Kennedy PT, Schurich A, Dunn C, et al. Blockade of immunosuppressive cytokines restores NK cell antiviral function in chronic hepatitis B virus infection. PLoS Pathog. 2010;6(12):e1001227. Epub 2010/12/29. doi: 10.1371/journal.ppat.1001227. PubMed PMID: 21187913; PubMed Central PMCID: PMC3003000.

3. Lv J, Jin Q, Sun H, Chi X, Hu X, Yan H, et al. Antiviral treatment alters the frequency of activating and inhibitory receptor-expressing natural killer cells in chronic hepatitis B virus infected patients. Mediators Inflamm. 2012;2012:804043. Epub 2013/01/11. doi: 10.1155/2012/804043. PubMed PMID: 23304062; PubMed Central PMCID: PMC3529875.

4. Zhao PW, Jia FY, Shan YX, Ji HF, Feng JY, Niu JQ, et al. Downregulation and altered function of natural killer cells in hepatitis B virus patients treated with entecavir. Clinical and experimental pharmacology & physiology. 2013;40(3):190-6. Epub 2013/01/03. doi: 10.1111/1440-1681.12048. PubMed PMID: 23278368.

5. Yan MX, Mao HT, Liu Q, Wang WQ, Li YQ. Elevated levels of serum soluble E-selectin in patients with chronic hepatitis B: Correlation with T lymphocyte subsets, NK cells and liver inflammation. Hepatol Res. 2006;35(2):111-7. Epub 2006/05/03. doi: 10.1016/j.hepres.2006.03.006. PubMed PMID: 16650799.

6. Sprengers D, van der Molen RG, Kusters JG, Hansen B, Niesters HG, Schalm SW, et al. Different composition of intrahepatic lymphocytes in the immune-tolerance and immune-clearance phase of chronic hepatitis B. Journal of medical virology. 2006;78(5):561-8. Epub 2006/03/24. doi: 10.1002/jmv.20576. PubMed PMID: 16555293.

7. Gu XB, Yang XJ, Wang D, Hua Z, Xu YQ, Lu ZH. Relationship between serum HBV DNA level and HBV-specific, nonspecific cytotoxic T lymphocytes and natural killer cells in patients with chronic hepatitis B. Chinese medical journal. 2009;122(18):2129-32. Epub 2009/09/29. PubMed PMID: 19781297.

8. Bonorino P, Ramzan M, Camous X, Dufeu-Duchesne T, Thelu MA, Sturm N, et al. Fine characterization of intrahepatic NK cells expressing natural killer receptors in chronic hepatitis B and C. J Hepatol. 2009;51(3):458-67. Epub 2009/07/15. doi: 10.1016/j.jhep.2009.05.030. PubMed PMID: 19596474.

9. Zhang Z, Zhang S, Zou Z, Shi J, Zhao J, Fan R, et al. Hypercytolytic activity of hepatic natural killer cells correlates with liver injury in chronic hepatitis B patients. Hepatology. 2011;53(1):73-85. Epub 2011/01/22. doi: 10.1002/hep.23977. PubMed PMID: 21254163; PubMed Central PMCID: PMC3767982.

10. Tjwa ET, van Oord GW, Hegmans JP, Janssen HL, Woltman AM. Viral load reduction improves activation and function of natural killer cells in patients with chronic hepatitis B. J Hepatol. 2011;54(2):209-18. doi: 10.1016/j.jhep.2010.07.009. PubMed PMID: 21095036.

11. Li W, Jiang Y, Wang X, Jin J, Qi Y, Chi X, et al. Natural Killer p46 Controls Hepatitis B Virus Replication and Modulates Liver Inflammation. PLoS One. 2015; 10: e0135874.

12. Zhao J, Li Y, Jin L, Zhang S, Fan R, Sun Y, et al. Natural killer cells are characterized by the concomitantly increased interferon-gamma and cytotoxicity in acute resolved hepatitis B patients. PLoS One. 2012;7(11):e49135. Epub 2012/11/08. doi: 10.1371/journal.pone.0049135. PubMed PMID: 23133672; PubMed Central PMCID: PMC3486810.

13. Li F, Wei H, Wei H, Gao Y, Xu L, Yin W, et al. Blocking the natural killer cell inhibitory receptor NKG2A increases activity of human natural killer cells and clears hepatitis B virus infection in mice. Gastroenterology. 2013;144(2):392-401. doi: 10.1053/j.gastro.2012.10.039. PubMed PMID: 23103614.

14. Sun C, Fu B, Gao Y, Liao X, Sun R, Tian Z, et al. TGF-beta1 down-regulation of NKG2D/DAP10 and 2B4/SAP expression on human NK cells contributes to HBV persistence. PLoS Pathog. 2012;8(3):e1002594. doi: 10.1371/journal.ppat.1002594. PubMed PMID: 22438812; PubMed Central PMCID: PMC3305436.

15. Lunemann S, Malone DF, Hengst J, Port K, Grabowski J, Deterding K, et al. Compromised function of natural killer cells in acute and chronic viral hepatitis. The Journal of infectious diseases. 2014;209(9):1362-73. doi: 10.1093/infdis/jit561. PubMed PMID: 24154737.

16. Li Y, Wang JJ, Gao S, Liu Q, Bai J, Zhao XQ, et al. Decreased peripheral natural killer cells activity in the immune activated stage of chronic hepatitis B. PLoS One. 2014;9(2):e86927. Epub 2014/02/13. doi: 10.1371/journal.pone.0086927. PubMed PMID: 24520324; PubMed Central PMCID: PMC3919705.

17. Tjwa ET, Zoutendijk R, van Oord GW, Biesta PJ, Verheij J, Janssen HL, et al. Intrahepatic natural killer cell activation, but not function, is associated with HBsAg levels in patients with HBeAg-negative chronic hepatitis B. Liver Int. 2014;34(3):396-404. doi: 10.1111/liv.12272. PubMed PMID: 23890390.

18. Conroy MJ, Mac Nicholas R, Grealy R, Taylor M, Otegbayo JA, O'Dea S, et al. Circulating CD56dim natural killer cells and CD56+ T cells that produce interferon-gamma or interleukin-10 are expanded in asymptomatic, E antigen-negative patients with persistent hepatitis B virus infection. Journal of viral hepatitis. 2015;22(3):335-45. doi: 10.1111/jvh.12299. PubMed PMID: 25186004.

19. Zheng Q, Zhu YY, Chen J, Ye YB, Li JY, Liu YR, et al. Activated natural killer cells accelerate liver damage in patients with chronic hepatitis B virus infection. Clin Exp Immunol. 2015;180(3):499-508. doi: 10.1111/cei.12597. PubMed PMID: 25639451; PubMed Central PMCID: PMC4449778.
