# Supplementary material for: Altered Immune Profiles of Natural Killer Cells in Chronic Hepatitis B Patients: A Systematic Review and Meta-Analysis
Source: PLoS One. 2016 Aug 11;11(8):e0160171. doi: 10.1371/journal.pone.0160171 (PMC4981347; doi:10.1371/journal.pone.0160171)
Supplement: S1 Table — (DOC) [file pone.0160171.s005.doc]

**S1 Table.** **Assessment of study quality.**

| First author, year | Type | Selection | Comparability | Exposure /Outcome | Total |
| --- | --- | --- | --- | --- | --- |
| Yan, 2006 | Case control | ** | ** | ** | ****** |
| Spreengers, 2006 | Case control | ** | ** | ** | ****** |
| Oliviero, 2009 | Case control | ** | ** | ** | ****** |
| Gu, 2009 | Case control | *** | ** | ** | ******* |
| Bonorino, 2009 | Case control | *** | * | ** | ****** |
| Peppa, 2010 | Case control | *** | ** | ** | ******* |
| Zhang, 2011 | Case control | *** | ** | ** | ******* |
| Tjwa, 2011 | Case control | ** | ** | ** | ****** |
|  | Cohort | *** | ** | ** | ******* |
| Lv, 2012 | Cohort | *** | ** | ** | ******* |
| Zhao J, 2012 | Case control | ** | ** | ** | ****** |
| Zhao P, 2012 | Cohort | *** | ** | ** | ******* |
| Li, 2012 | Case control | ** | ** | ** | ******* |
| Sun, 2012 | Case control | ** | ** | ** | ****** |
| Lunemann, 2013 | Case control | ** | ** | ** | ****** |
| Li, 2014 | Case control | ** | ** | ** | ****** |
| Tjwa, 2014 | Cohort | *** | ** | ** | ******* |
| Conroy, 2014 | Case control | *** | ** | ** | ******* |
| Li, 2015 | Case control | ** | ** | ** | ****** |
| Zheng, 2015 | Case control | ** | ** | ** | ****** |
