# Supplementary material for: Altered Immune Profiles of Natural Killer Cells in Chronic Hepatitis B Patients: A Systematic Review and Meta-Analysis
Source: PLoS One. 2016 Aug 11;11(8):e0160171. doi: 10.1371/journal.pone.0160171 (PMC4981347; doi:10.1371/journal.pone.0160171)
Supplement: S2 Table — (DOC) [file pone.0160171.s006.doc]

**S2 Table.** **Sources of heterogeneity of 14 selected studies of peripheral NK cells.**

|  | Coefficient | 95% CI | P |
| --- | --- | --- | --- |
| ALT | –1.405647 | –2.709592 to –0.1017016 | 0.037 |
| HBV–DNA levels | –0.2572969 | –0.7976867 to 0.2830928 | 0.320 |
| Donor source | 0.9985603 | 0.9929757 to 1.004176 | 0.588 |
| Male percentage | –0.335123 | –0.5168006 to 0.4497759 | 0.882 |
| Age | 0.9822493 | 0.8615872 to 1.11981 | 0.772 |
| Case sample size | 0.0026711 | –0.284152 to 0.337574 | 0.856 |
| Publication year | –0.1674658 | –0.4451156 to 0.1101841 | 0.215 |
