# Supplementary material for: Altered Immune Profiles of Natural Killer Cells in Chronic Hepatitis B Patients: A Systematic Review and Meta-Analysis
Source: PLoS One. 2016 Aug 11;11(8):e0160171. doi: 10.1371/journal.pone.0160171 (PMC4981347; doi:10.1371/journal.pone.0160171)
Supplement: S3 Table — (A) Quantitative Data of pooled frequency of peripheral NK cells on Bias. (B) Quantitative Data of pooled frequency of CD107a degranulation of NK cells on Bias. (C) Quantitative Data of pooled frequency of IFNγ production of NK cells on Bias. (DOC) [file pone.0160171.s007.doc]

**S3A Table.** **Quantitative Data of pooled frequency of peripheral NK cells on Bias.**

| Number of studies | Kendall’s Scores |  | SD Score |  | Z | P＞|Z| |
| --- | --- | --- | --- | --- | --- | --- |
| Begg’s test 14 | –35 |  | 20.21 |  | 1.68 | 0.092 |
| Std. Eff. | Coeff. | SE | *t* | *P >|t|* | 95% CI |  |
| Egger’s test |  |  |  |  |  |  |
| Slope | 1.119205 | 1.128504 | 0.99 | 0.339 | –1.31878 to 3.55719 |  |
| Bias | –5.026991 | 4.018058 | –1.25 | 0.233 | –13.70748 to 3.653495 |  |

Begg’s test was not significant (Z = 1.68, *P* = 0.092) and this indicated that there was no potential publishing bias. Similar results were obtained after Egger’s test (*t* = –1.25, *P* = 0.233).

**S3B Table.** **Quantitative Data of pooled frequency of CD107a degranulation of NK cells on Bias.**

| Number of studies | Kendall’s Scores |  | SD Score |  | Z | P＞|Z| |
| --- | --- | --- | --- | --- | --- | --- |
| Begg’s test 9 | 18 |  | 9.59 |  | 1.77 | 0.076 |
| Std. Eff. | Coeff. | SE | *t* | *P >|t|* | 95% CI |  |
| Egger’s test |  |  |  |  |  |  |
| Slope | –1.678603 | 0.9599323 | –1.75 | 0.124 | –3.948482 to 0.5912764 |  |
| Bias | 6.178165 | 2.926374 | 2.11 | 0.073 | –0.741609 to 13.09794 |  |

Begg’s test was not significant (Z = 1.77, P = 0.076) and this indicated that there was no potential publishing bias. Similar results were obtained after Egger’s test (t = 2.11, P = 0.073).

**S3C Table.** **Quantitative Data of pooled frequency of IFN-γ production of NK cells on Bias.**

| Number of studies | Kendall’s Scores |  | SD Score |  | Z | P＞|Z| |
| --- | --- | --- | --- | --- | --- | --- |
| Begg’s test 8 | 4 |  | 8.08 |  | 0.37 | 0.711 |
| Std. Eff. | Coeff. | SE | *t* | *P >|t|* | 95% CI |  |
| Egger’s test |  |  |  |  |  |  |
| Slope | –0.4700695 | 0.6658243 | –0.71 | 0.507 | –2.099283 to 1.159144 |  |
| Bias | 1.048159 | 2.506257 | 0.42 | 0.690 | –5.08443 to 7.180749 |  |

Begg’s test was not significant (Z = 0.37, P = 0.711) and this indicated that there was no potential publishing bias. Similar results were obtained after Egger’s test (t = 0.42, P = 0.690).
